# Examination of Endoscopic Marsupialization of Vocal Fold Cyst Under Topical Anesthesia

**DOI:** 10.7759/cureus.85949

**Published:** 2025-06-13

**Authors:** Ray Motohashi, Ryoji Tokashiki, Yusuke Shoji, Ujimoto Konomi, Kiyoaki Tsukahara

**Affiliations:** 1 Otorhinolaryngology - Head and Neck Surgery, Tokyo Medical University, Tokyo, JPN; 2 Otolaryngology, Shinjuku Voice Clinic, Tokyo, JPN

**Keywords:** cathelin needle, day surgery, endoscopic laryngeal needle surgery, endoscopic marsupialization, laryngoplasty, office-based interventions, thyrohyoid approach, topical anesthesia, vocal fold cyst

## Abstract

Endoscopic marsupialization of vocal fold cysts under topical anesthesia was evaluated in 107 patients to determine its efficacy and outcomes. The recurrence rate was 4.7%, with all recurrent cases successfully treated with reoperation. Significant improvements in Voice Handicap Index (VHI), Maximum Phonation Time (MPT), and Pitch Range were observed at two to four weeks postoperatively and maintained at six months. This minimally invasive procedure, which avoids general anesthesia, demonstrated comparable recurrence rates to traditional methods while offering superior cost-effectiveness and faster recovery. These findings suggest that endoscopic marsupialization is an effective and patient-friendly alternative for managing vocal fold cysts.

## Introduction

Vocal fold cysts are benign laryngeal lesions characterized by retention cysts or epidermoid cysts within the vocal fold mucosa. These lesions disrupt the vibration of the vocal fold mucosa during phonation, leading to voice disorders. Clinical symptoms typically include hoarseness and vocal strain. Diagnosis is established through laryngeal fiberscopy, which reveals submucosal elevations, and stroboscopy, which shows reduced mucosal vibration.

Standard treatment involves complete excision of the cyst to prevent recurrence, typically performed under general anesthesia using laryngeal microsurgery. However, complete excision can lead to extensive tissue trauma, prolonged recovery of mucosal vibration, and in some cases, permanent voice deterioration due to scarring. For retention cysts, attempts to excise the cyst wall may cause significant mucosal damage, particularly if the cyst collapses during surgery [1]. Recently, marsupialization under general anesthesia, where the cyst wall is incised and removed, has been reported to yield low recurrence rates and good voice outcomes [2,3]. Based on this approach, our team has utilized endoscopic laryngeal needle surgery (ELNS) under topical anesthesia for marsupialization of vocal fold cysts since 2010 [4,5]. In 2017, we reported on 48 cases, demonstrating no recurrence rate and significant voice improvement within one month postoperatively [6]. This study expands on these findings, analyzing 107 cases treated with ELNS under topical anesthesia to evaluate the method’s recurrence rate and voice outcomes.

## Materials and methods

This study was approved by the Institutional Review Board of Tokyo Medical University (approval number: T2023-0206), and the need for informed consent was waived owing to its retrospective nature.

Subjects

This study included 107 cases of vocal fold cysts treated with outpatient ELNS under topical anesthesia between July 2010 and June 2018 at Tokyo Medical University Hospital and Shinjuku Voice Clinic.

Preparation

**Figure 1:**
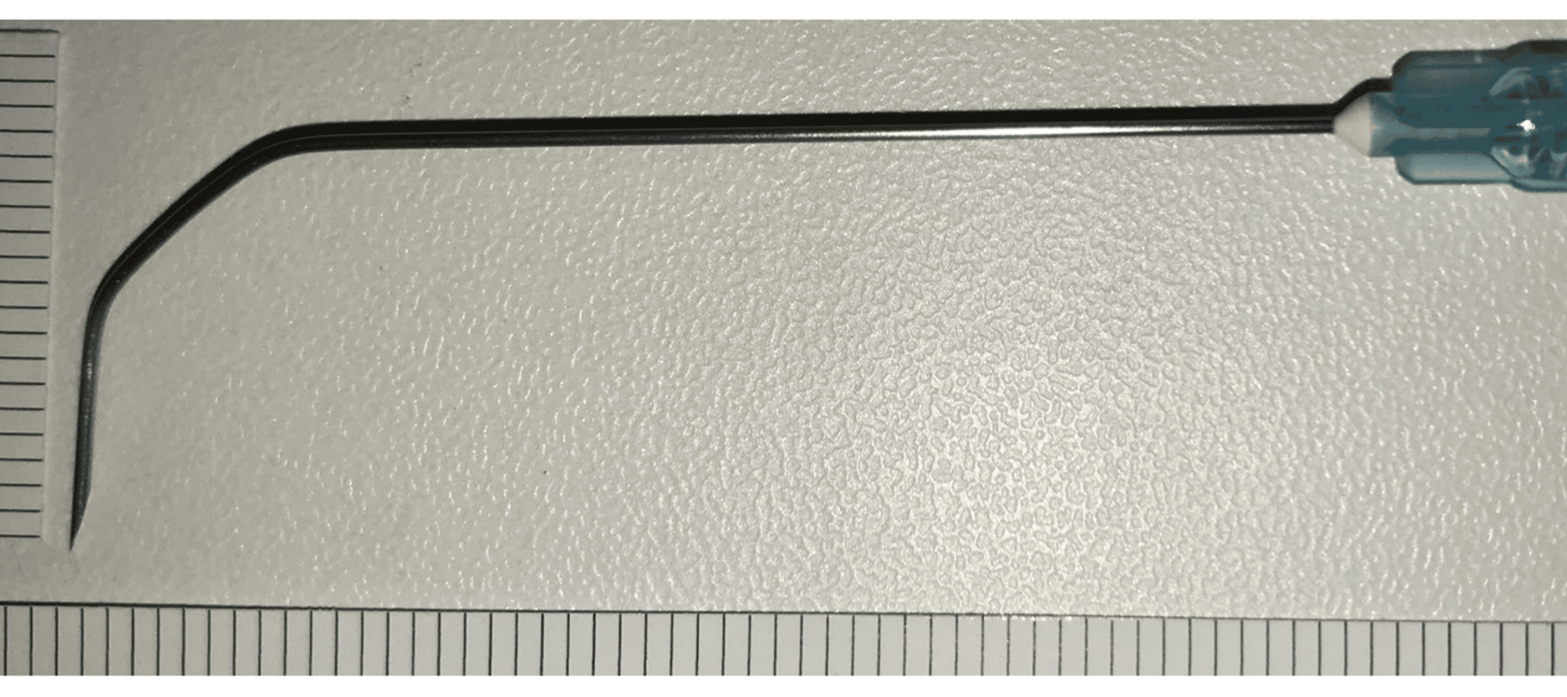
A double-bent 60 mm Cathelin needle A 60 mm, 23-G Cathelin needle bent at 45° by hand at two sites. The figure is adapted from Motohashi [5], with permission obtained for its use.

Topical anesthesia was achieved using a 4% lidocaine nebulizer, followed by spraying 4% lidocaine onto the tongue base, epiglottis, and vocal folds through a suction-equipped laryngeal fiberscope. A 23-G, 60-mm Cathelin needle was bent at two points (1-1.5 cm and 2-3 cm from the tip) at 45-degree angles, and a 1.0- or 2.5-mL syringe was attached as a handle (Figure 1).

Surgical procedure

**Figure 2:**
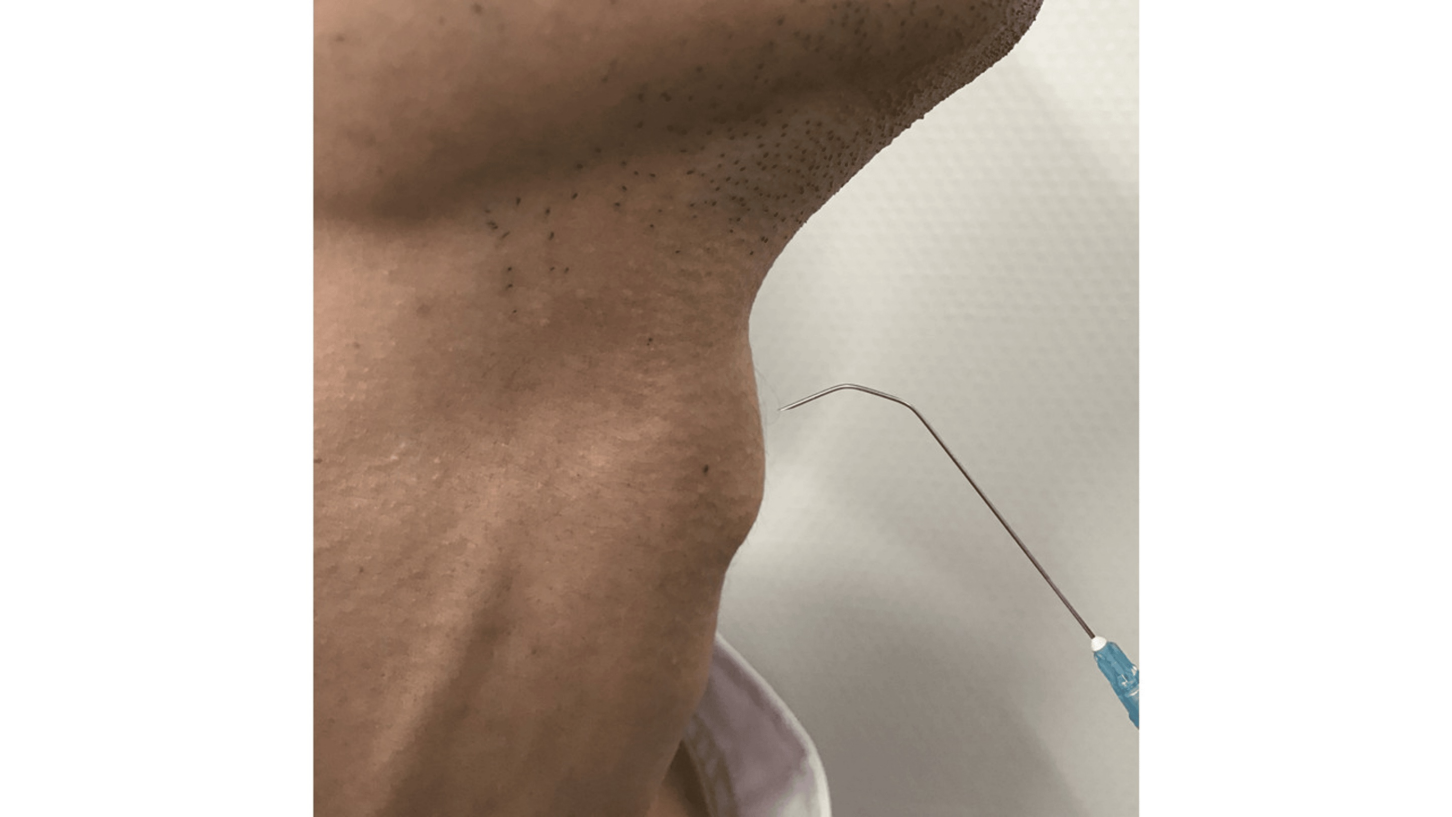
Puncture procedure The prepared Cathelin needle was inserted above the superior thyroid notch. The figure is adapted from Motohashi [5], with permission obtained for its use.

With the patient seated, a laryngeal fiberscope was used to observe the larynx. The prepared Cathelin needle was inserted percutaneously just above the thyroid notch, aimed slightly downward (Figure 2).

**Figure 3:**
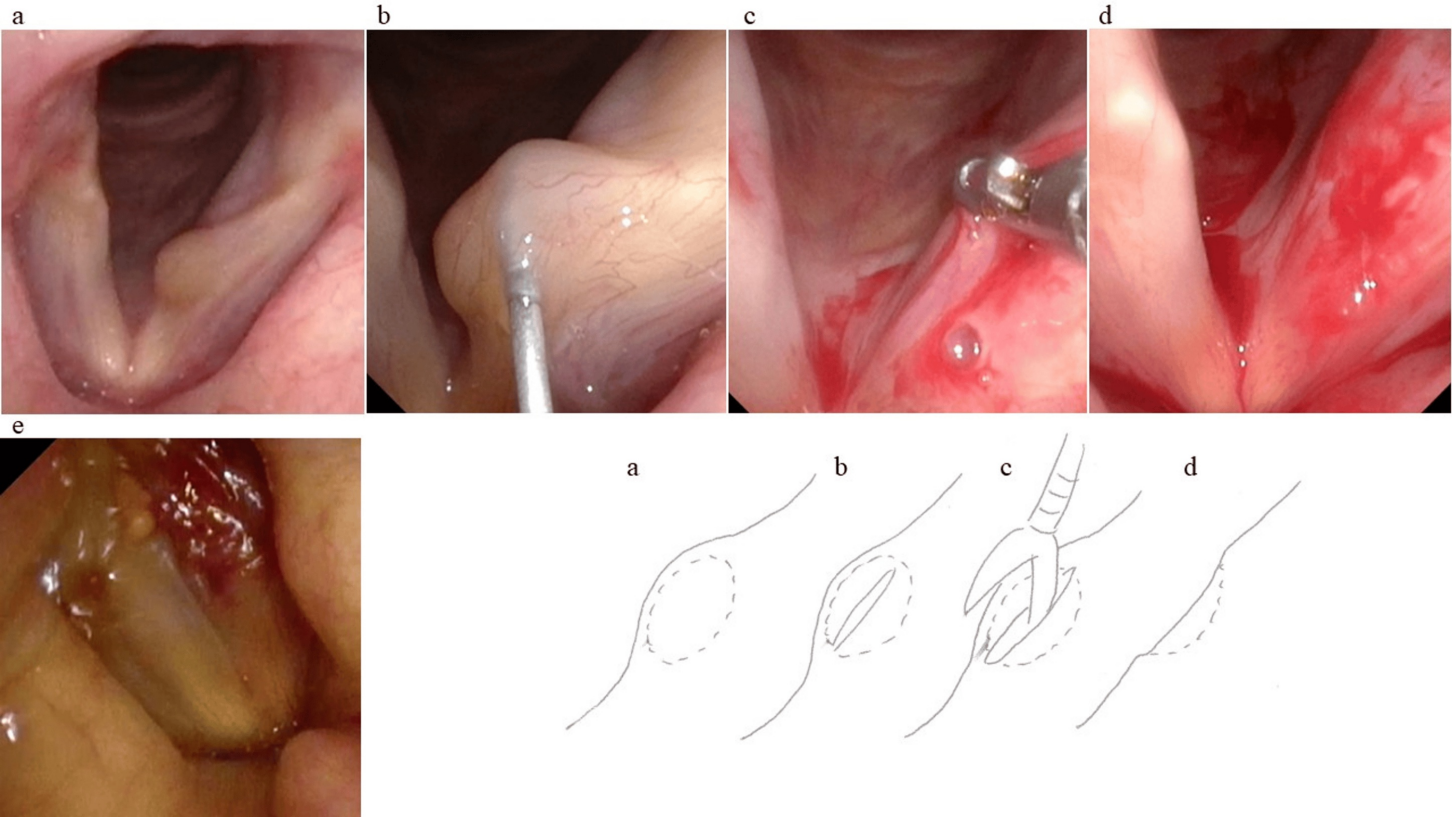
Surgical procedure and corresponding schematic (a) Preoperative vocal fold cyst. (b) The cyst was incised with a Cathelin needle, and the incision was enlarged. (c) Subsequently, forceps were introduced through a suction-equipped fiberscope to excise the outer mucosa of the cyst wall as a single unit. (d) Postoperative vocal fold findings. (e) The procedure concluded upon confirming mucosal wave restoration via stroboscopy. Schematic illustration created by the author based on the present case.

The needle tip was advanced beneath the epiglottic tubercle to incise the cyst wall, and cyst contents were aspirated. Subsequently, forceps were introduced through the suction-equipped fiberscope to excise the outer mucosa of the cyst wall as a single unit. The procedure concluded upon confirming mucosal wave restoration via stroboscopy (Figure 3).

Vocal function evaluation

Recurrence and time to recurrence were assessed during regular follow-up. Of the 107 cases, 65 underwent voice function tests at their first postoperative visit (one month), and 45 at six months postoperatively. Statistical analysis of preoperative and postoperative parameters included the Voice Handicap Index (VHI), Maximum Phonation Time (MPT), Pitch Range, Shimmer, Jitter, and Noise-to-Harmonic Ratio (NHR). MPT was measured by instructing the patient to sustain the vowel “a” at a comfortable speech volume for as long as possible. The best out of three attempts was selected. The MFR was measured by producing a sustained vowel /a/ or /u/ into a cylindrical mouthpiece. Aerodynamic assessments, including MPT and MFR evaluations, were performed using a phonation analyzer (PS-77E; Nagashima Medical Instruments Co., Tokyo, Japan). Acoustic analyses evaluated Jitter, Shimmer, and NHR using a Computerized Speech Lab (CSL) 4500 (KayPENTAX, Lincoln Park, NJ) and a Multi-Dimensional Voice Program (Model 5105, KayPENTAX, Lincoln Park, NJ).

Statistical analysis

Wilcoxon’s signed-rank test was used for comparisons between pre- and post-treatment values, and statistical significance was set at p < 0.05.

## Results

Patient characteristics

The cohort included 63 males and 44 females, aged 21 to 84 years (mean age: 50 years). Cysts were located on the left vocal fold in 61 cases and on the right in 46. There were only six cases of epidermoid cysts. The surgical procedure took less than 10 minutes in all cases.

Recurrence rate and progression

Five cases (4.7%) experienced recurrence within two weeks to eight months postoperatively, all of which underwent reoperation. All recurrent cases were retention cysts. Four of these patients showed no recurrence during follow-up periods of five to 16 months, while one was lost to follow-up.

Changes in speech parameters from preoperative to postoperative one month

**Figure 4:**
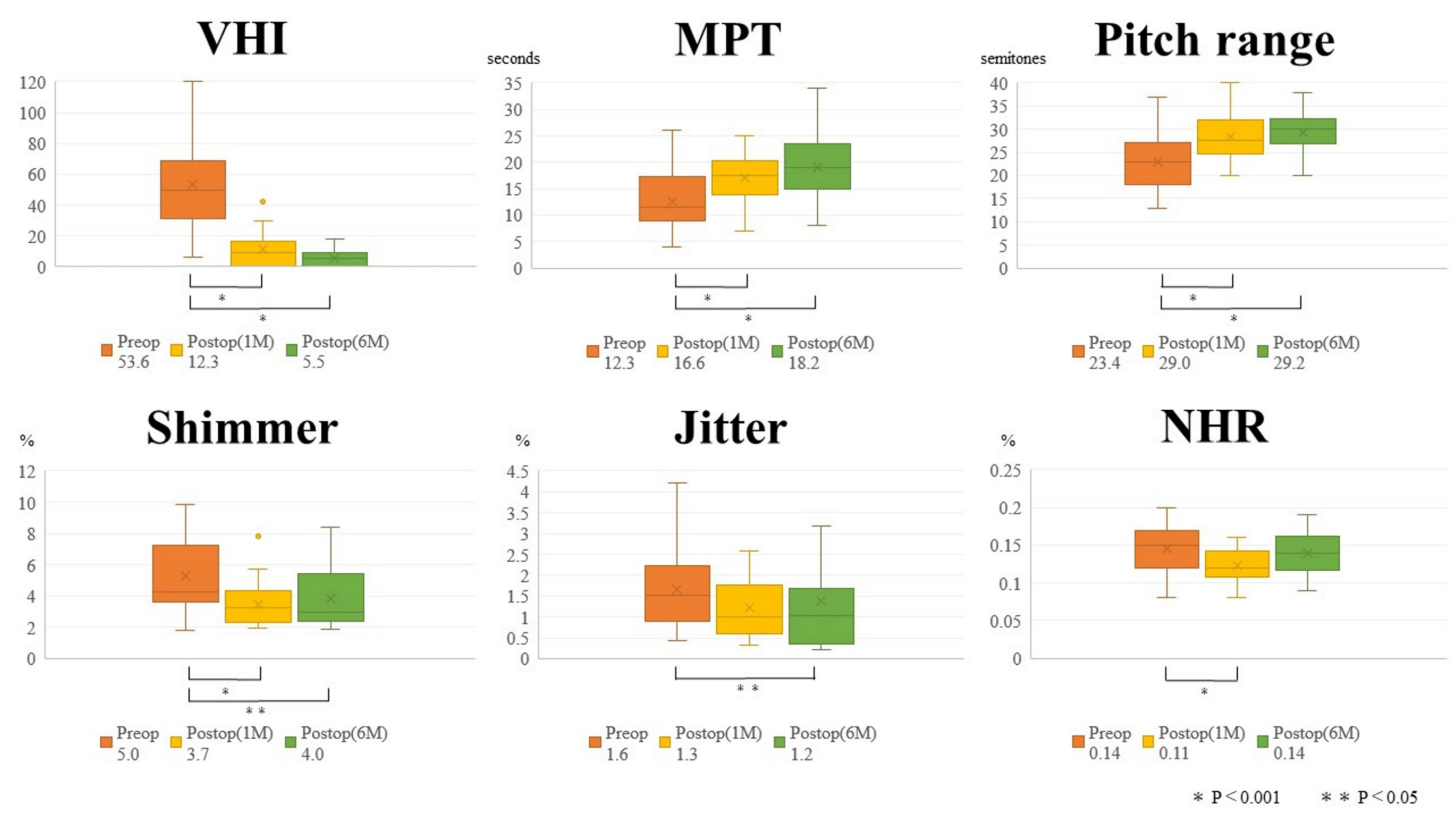
Preoperative and postoperative changes in speech parameters Changes in speech parameters from preoperative to postoperative one month: Significant improvements were observed in Voice Handicap Index (VHI) (p < 0.001), Maximum Phonation Time (MPT) (p < 0.001), Pitch Range (p < 0.001), Shimmer (p < 0.001), and Noise-to-Harmonic Ratio (NHR) (p < 0.001). No significant improvement was noted in Jitter (p = 0.086). Changes in speech parameters from preoperative to postoperative six months: Significant improvements persisted in VHI (p < 0.001), MPT (p < 0.001), Pitch Range (p < 0.001), Shimmer (p < 0.05), and Jitter (p < 0.05), with a non-significant trend toward improvement in NHR (p = 0.114).

Significant improvements were observed in VHI (53.6 ± 28.7 vs. 12.3 ± 11.4, p < 0.001), MPT (12.3 ± 5.6 vs. 16.6 ± 5.2 seconds, p < 0.001), Pitch Range (23.4 ± 6.2 vs. 29.0 ± 4.5 semitones, p < 0.001), Shimmer (5.0 ± 2.0 vs. 3.7 ± 1.4 %, p < 0.05), and NHR (0.14 ± 0.03 vs. 0.12 ± 0.02 %, p < 0.001). No significant improvement was noted in Jitter (1.6 ± 0.9 vs. 1.3 ± 0.7 %, p = 0.086) (Figure 4).

Changes in speech parameters from preoperative to postoperative six months

Significant improvements persisted in VHI (53.6 ± 28.7 vs. 5.5 ± 5.5, p < 0.001), MPT (12.3 ± 5.6 vs. 18.2 ± 6.3 seconds, p < 0.001), Pitch Range (23.4 ± 6.2 vs. 29.2 ± 4.7 semitones, p < 0.001), Shimmer (5.0 ± 2.0 vs. 4.0 ± 1.7%, p < 0.05), and Jitter (1.6 ± 0.9 vs. 1.2 ± 0.9 %, p < 0.05), with a non-significant trend toward improvement in NHR (0.14 ± 0.03 vs. 0.14 ± 0.02 %, p = 0.114) (Figure 4).

## Discussion

The treatment for vocal cord cysts is generally total removal under general anesthesia using laryngomicrosurgery using the microflap method reported by Sataloff et al. [7] and Courey et al. [8,9], since there is a risk of recurrence if the cyst is not completely removed. The advantage of these methods is the prevention of recurrence by completely removing the cyst's membrane. However, complete excision can result in a larger surgical area than the cyst itself, prolong recovery of mucosal vibration, and in some cases, cause permanent voice deterioration due to scarring [7]. Furthermore, recurrence may still occur even with complete excision [10]. Recently, reports of marsupialization under general anesthesia or in-office endoscopic marsupialization under topical anesthesia have emerged.

Marsupialization under general anesthesia, described by Tai et al. [2]. Cyst enucleation is the ideal procedure, but cyst rupture often occurs. Marsupialization is useful due to its simplicity, minimal tissue damage, early functional improvement, and low recurrence rate [2]. In cases where the cyst wall is ruptured, overzealous attempts to remove residual cyst walls can cause irreversible scarring. Chang et al. demonstrated that marsupialization, performed under general anesthesia in 21 cases, achieved comparable voice improvement to complete excision with only one recurrence [3]. Iida et al. reported on in-office fenestration surgery for vocal fold cysts under topical anesthesia, showing low recurrence rates, satisfactory voice outcomes, and reduced financial and time burdens for patients [11].

Our team has employed ELNS for marsupialization of vocal fold cysts under topical anesthesia since May 2010. In 2017, we reported on 48 cases, highlighting early voice improvement and no recurrences [5]. A key advantage of ELNS is its ability to allow real-time monitoring of vocal fold vibration during surgery. The immediate confirmation of surgical efficacy via stroboscopy is possible. Traditional transoral approaches under topical anesthesia often induce pharyngeal reflexes, causing discomfort for patients and making surgery completion difficult in about 10% of cases [12]. However, our percutaneous approach avoids triggering such reflexes, and no cases in our study were terminated due to this issue. Additionally, ELNS is feasible for patients who cannot tolerate general anesthesia or in cases where laryngeal exposure is challenging under general anesthesia.

From a cost perspective, laryngeal microsurgery under general anesthesia generally requires hospitalization, which incurs significant expenses and time commitments. Patients undergoing general anesthesia also face associated risks, making it less suitable for elderly individuals or those with systemic comorbidities. In contrast, ELNS under topical anesthesia eliminates the need for hospitalization, significantly reducing costs, approximately one-fifth compared to general anesthesia procedures. However, limitations include the inability to perform complete cyst excision or suturing and challenges with patients exhibiting strong reflexes or a history of asthma.

Recurrence

In our study, five of 107 cases (4.7%) experienced recurrence within two weeks to eight months postoperatively. All recurrences were treated with repeat ELNS, with no further recurrence in four cases during follow-up; one case did not return for a follow-up visit. Past studies have reported recurrence rates of 2.2%-6.7% for surgeries under general anesthesia [1,11,13-16]. While complete excision is aimed at preventing recurrence, cyst wall rupture may necessitate marsupialization, which has been shown to have low recurrence rates even when the cyst wall is not completely excised [11]. In the case of retention cysts, it has been reported that the cyst wall was damaged in 91 of 116 cases, but all 91 of these damaged cases were marsupialization rather than completely removed, and recurrence was reported in 10 cases (1.1%) [17], showing a low recurrence rate with marsupialization. Iida et al. reported three cases (7.5%) recurrence in their series of 40 cases using in-office endoscopic excision under topical anesthesia and suggested that this method could be a viable alternative to general anesthesia procedures, as the recurrence rates are comparable [11]. Similarly, our study demonstrates that ELNS achieves recurrence rates comparable to those of general anesthesia-based procedures while maintaining good voice outcomes.

Voice function evaluation

Significant improvements in VHI, MPT, Pitch Range, Shimmer, and Jitter were observed at the first postoperative follow-up (two to four weeks), with trends toward improvement in NHR. Although no prior reports exist on recovery time for vocal function following cyst surgery, the rapid recovery observed in our awake procedure allows immediate intraoperative confirmation of improvement. Evaluations at six months postoperatively confirmed sustained improvements in voice parameters, supporting the long-term efficacy of this technique.

Tanaka et al. reported no significant differences in voice outcomes between complete excision and marsupialization groups [18]. Our findings align with these results, demonstrating early and sustained voice improvements after ELNS. Hsu et al. [1] and Tibbetts et al. [15] have reported similar improvements with surgeries under general anesthesia, further supporting the efficacy of marsupialization as a surgical option.

However, this study has several limitations. First, it is a retrospective study conducted at two centers and, thus, may be subject to selection bias. Second, not all patients underwent long-term follow-up, and only 45 of the 107 cases received voice evaluations at six months, limiting the generalizability of the long-term results. Third, comparisons with general anesthesia procedures were based on previous reports rather than a direct control group within the study.

## Conclusions

The necessity of complete excision for vocal fold cysts remains a topic of debate. Recurrence can occur even with complete excision, suggesting that marsupialization may be a viable alternative, particularly given the favorable voice outcomes observed in this study. In our cases, repeat ELNS was effective for treating recurrences, with no further recurrence observed. Continued evaluation of recurrence cases may help define the limitations and indications of this technique.

While complete excision remains the standard treatment for vocal fold cysts, our findings indicate that endoscopic marsupialization under topical anesthesia achieves comparable recurrence rates and early voice improvement. This minimally invasive, cost-effective procedure offers a valuable alternative for managing vocal fold cysts, particularly in patients for whom general anesthesia is contraindicated or undesirable.
